# Use of Cold Water in Erysipelas

**Published:** 1846-05

**Authors:** 


*The Use of Cold Water in Erysipelas.*—The cold affusion is not to be employed in all cases, nor in any without great consideration; for very serious consequences may result from its injudicious adoption. According to my experience, it is when the exanthem exhibits a livid or bluish color; when the affected skin is little swollen, but is dry and is the seat of a burning prickling heat; when the temperature of the body is unequal, the extremities being perhaps cold, while the head is hot; and when the accompanying fever is of a nervous typhoid type, that the cold affusion is sometimes of great service; indeed, the only remedy which holds out any prospect of decided advantage. When this is the case, we observe, after its employment, that the skin becomes more turgescent, that the color of the eruption is more decidedly red than it was before, that the delirium abates, and the tongue is moister —changes, which indicate that the remedy need not be repeated. If, however, the former state of things returns, it will be necessary again to have recourse to the affusion. I have seen another form of erysipelas, in which the cold affusion has been employed with signal advantage: this form prevailed epidemically in the hospital at Zurich in the year 1836. The exanthem did not come out upon the surface fully or regularly; there were only patches scattered here and there; and these appeared for a short while, and then vanished. At the same time, the sensorium was much disturbed. A few of the patients lay in a state of stupor; but in most there was a strange confusion of mind, so that they left their beds, and went about the wards, as if they were going to their ordinary work. Some of them squatted themselves down upon the floor, fancying that they were on the night-stool, or in the water-closet. In most instances, the pulse was but little excited, except towards evening; in some it was below the natural standard. After the use of the cold affusion, the exanthem came out much more freely, the face became redder and more swollen, and altogether there was a more distinct vascular action than had existed before. In most of the patients, the effects of the douche was of short duration; it required to be repeated in the course of a few hours, and even a third time. There is still another form of the disease in which the remedy has been used with marked benefit. As we saw in our present patient that the erysipelatous affection stood still, and that the skin, under the exfoliating integument, exhibited a new redness, so we not unfrequently have occasion to observe a similar phenomenon in elderly persons, who are affected with a constant erysipelas of the face; the red patches upon the point of the nose or over the zygomatic arch, for example, being surrounded with a red ring, and presenting a shining surface. If in this state the head becomes affected, and if the patient is delirious, has a rapid weak pulse, a dry coated tongue, and the stomach is perhaps irritable to vomiting, I believe that there is no remedy that promises so much benefit as the judicious employment of the cold affusion. The old physicians in such cases recommended an emetic in the first instance, and afterwards the use of camphor and such-like stimulants; but I greatly prefer the above treatment.—*Schoenlein's Clinical Lectures.—Medico-Chirurgical Review.*

